# Crystal Structure of a Complex of DNA with One AT-Hook of HMGA1

**DOI:** 10.1371/journal.pone.0037120

**Published:** 2012-05-16

**Authors:** Elsa Fonfría-Subirós, Francisco Acosta-Reyes, Núria Saperas, Joan Pous, Juan A. Subirana, J. Lourdes Campos

**Affiliations:** 1 Departament d'Enginyeria Química, Universitat Politècnica de Catalunya, Barcelona, Spain; 2 Plataforma Automatitzada de Cristal.lografia, Institut de Recerca Biomèdica de Barcelona, PCB-CSIC, Barcelona, Spain; International Centre for Genetic Engineering and Biotechnology, Italy

## Abstract

We present here for the first time the crystal structure of an AT-hook domain. We show the structure of an AT-hook of the ubiquitous nuclear protein HMGA1, combined with the oligonucleotide d(CGAATTAATTCG)_2_, which has two potential AATT interacting groups. Interaction with only one of them is found. The structure presents analogies and significant differences with previous NMR studies: the AT-hook forms hydrogen bonds between main-chain NH groups and thymines in the minor groove, DNA is bent and the minor groove is widened.

## Introduction

**Figure 1:**
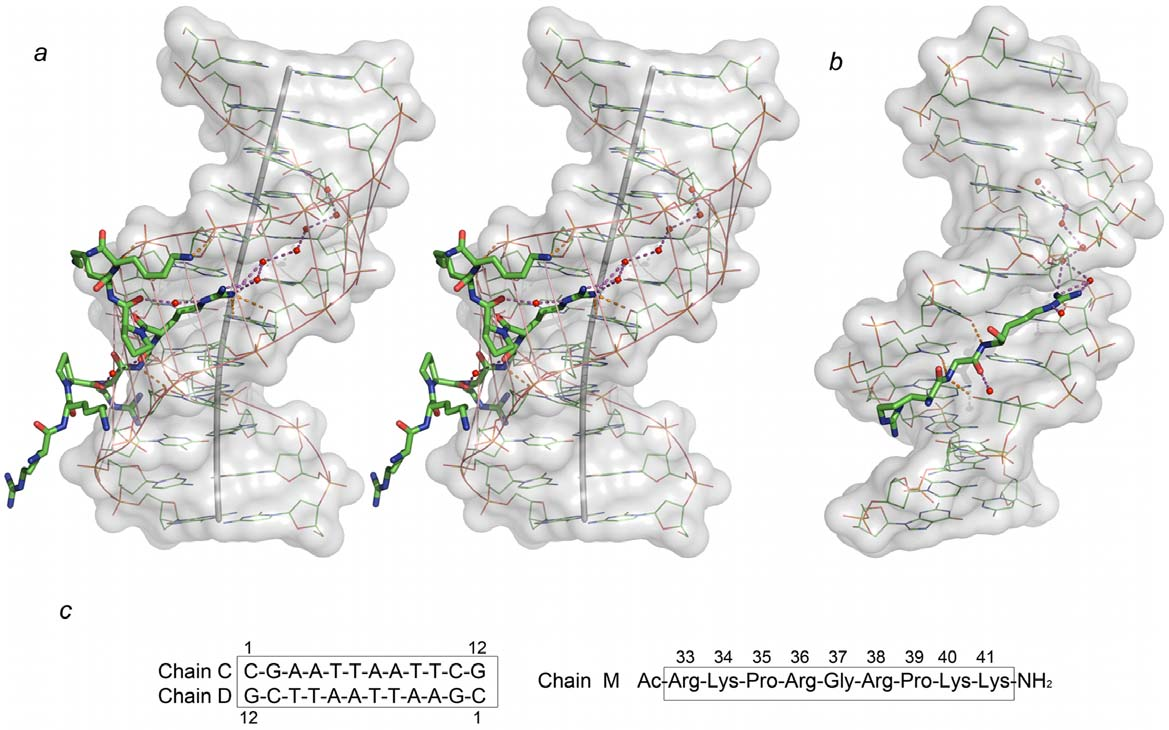
Stereoviews and composition of the AT-hook/DNA complex. **a** Stereoview of one AT-hook/DNA complex. The DNA is shown as a partially transparent object, drawn with Pymol. Its bent helical axis is shown in darker grey (calculated with CURVES+, available at http://gbio-pbil.ibcp.fr/Curves_plus/). Two water molecules which contribute to stabilize the structure are also represented, as well as waters in the spine of hydration. Virtual bonds between phosphates are indicated by thin red lines: they demonstrate a much wider minor groove in the region where the AT-hook is inserted. **b**. In this view only the elongated central region of the AT-hook (Arg-Gly-Arg) is shown. Hydrogen bonds between main chain peptide NH atoms (Gly37 and Arg38) and thymine oxygen atoms are also indicated. **c**. Numbering of the oligonucleotide and peptide residues used in this work. In order to facilitate comparison we have used the same numbering as reported for the DBD3 hook in the NMR structure [10].

High mobility group A proteins (HMGA, formerly called HMG-I/Y) are intrinsically disordered non-histone chromosomal proteins characterized by containing three DNA-binding domains, called AT-hooks, which preferentially bind to the minor groove of short stretches of AT-rich DNA [1]. By binding to differently spaced AT-rich DNA regions and/or direct interaction with several transcription factors, HMGAs regulate the expression of numerous genes. In this way they influence many normal biological processes including growth, proliferation, differentiation and death [2]–[4]. Alterations or abnormal expression of HMGA proteins have also been related to several pathological processes and metabolic disorders, including obesity, type 2 diabetes mellitus and cancer [5]–[7]. Thus, HMGA proteins have been proposed as therapeutic drug targets by several authors [7], [8]. The nature of the interaction was already determined by Reeves and Nissen [1], who used foot-printing methods. Further details on the interaction were later determined by NMR methods [9], [10]. From the structural point of view no results are available on the interaction of HMGA with DNA, as determined by crystallographic methods. Here we present the crystal structure of the complex of a DNA oligonucleotide with the third AT-hook (DBD3 in ref. 10) of the HMGA1 protein. Their sequence is given in Figure 1c.

**Figure 2:**
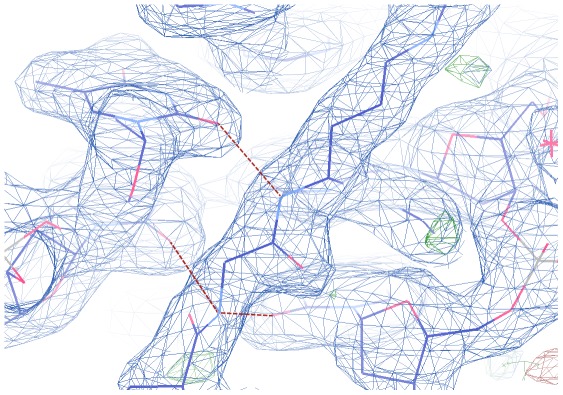
Electron density map (2F_o_-F_c_ at 1σ level) of a segment of the AT-hook in the minor groove of DNA. Hydrogen bonds between main chain peptide NH atoms (Gly37 and Arg38) and thymine oxygen atoms are indicated as red dashed lines.

**Figure 3:**
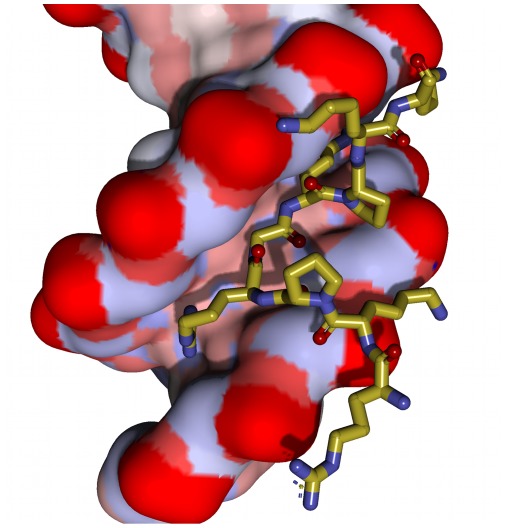
In this figure the DNA duplex is shown as space filling atoms with their van der Waals radii. The strong association of the inner PRGRP sequence with the duplex is clearly visible. The terminal basic amino acids (34, 40 and 41) interact with phosphates in the same duplex, with the exception of Arg33 which does not show any apparent interactions.

**Figure 4:**
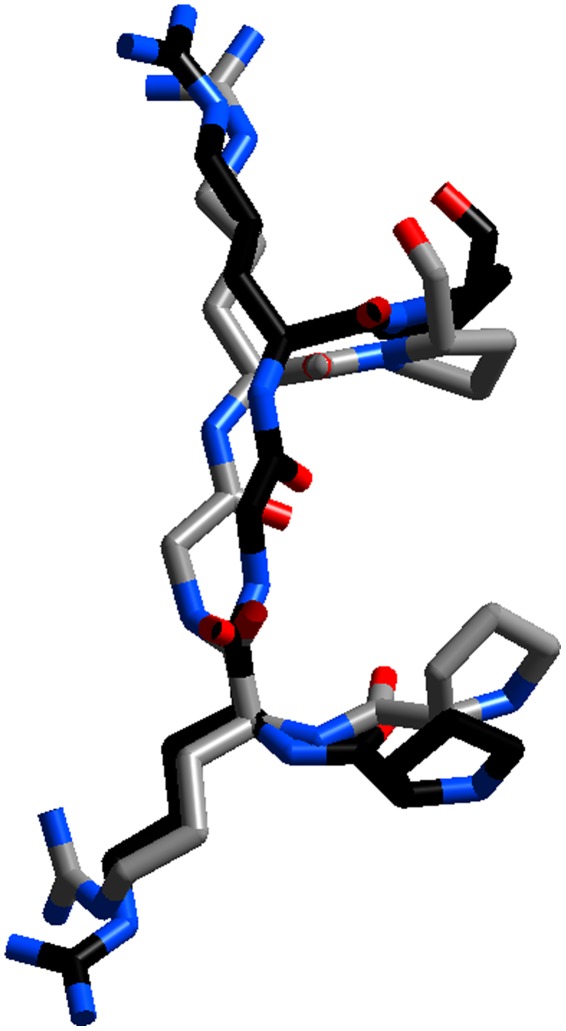
Comparison of the structures of the central PRGRP sequence of the AT-hook obtained by either X-ray diffraction (in grey, this work) or NMR [**10**]. The overall conformation is similar, but the X-ray structure has significant changes in the position of the main chain NH groups, which allow the formation of hydrogen bonds with minor groove thymine oxygen atoms of DNA. This figure has been prepared with Cerius2.

**Figure 5:**
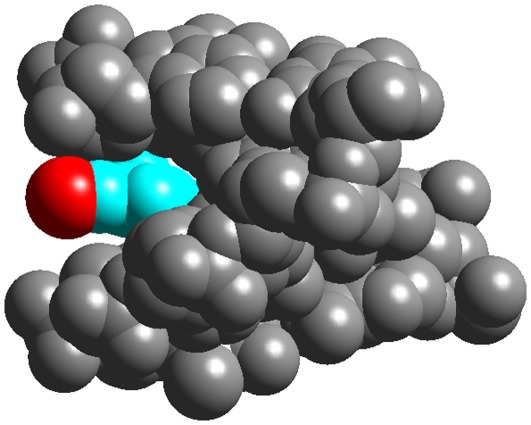
A model of the effect of methylation on Arg38 in the DBD3 AT-hook. Only the guanidinium group of Arg38 (in blue) is visible in this orientation. DNA is shown in grey. In this model the hydrogen bonds between the arginine guanidinium group and the minor groove of DNA are maintained, whereas the methyl group (in red) has an external position. In the side facing DNA there is no room for the methyl group, without disturbing the guanidinium-DNA hydrogen bonds.

**Table 1:** Crystal data and refinement statistics.

Wavelength (Å)	0.9795
Space grup	P21
Unit-cell parameters (Å, °)	a = 49.143, b = 66.855, c = 50.052, α = γ = 90 β = 111.4
Resolution range (Å)	40.00–2.27 (2.31–2.27)
Unique reflections	13163 (994)
Free *R*-factor reflections	740 (47)
Completeness (%)	98.73 (98.2)
Average Redundancy	7.5 (7.3)
[I/σ(I)]	2.2 (1.6)
R_merge_	0.166 (0.346)
Contents of asymmetric unit	4 DNA duplexes, 4 peptide molecules, 59 water molecules. Total: 2289 non-H atoms.
R_work_ a	0.225 (0.321)
R_free_ b	0.271 (0.387)
Mean *B* factor (Å^2^)	36.36
R.m.s.d. bonds (Å)	0.006
R.m.s.d. angles (°)	1.062

Values in parentheses are for the last shell.

a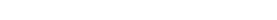.

b *R* factor of reflections used for cross validation in the refinement.

**Figure 6:**
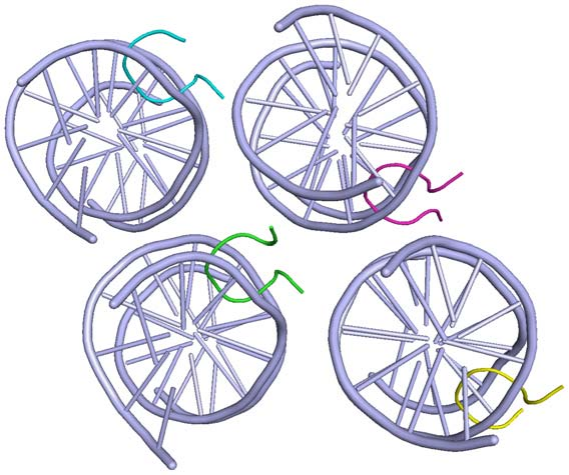
Simplified view of the asymmetric unit in the crystal. The two complexes at right are in the up orientation, as shown in Figure 1 in the main text. The two complexes at left are in the opposite orientation, with the peptide running down. In the crystal each complex generates a continuous column of identical complexes related by a screw axis. A detailed analysis of the packing interactions will be reported elsewhere.

As mentioned above, the HMGA1 protein contains three AT-hooks. All of them are formed by a conserved core sequence, Pro-Arg-Gly-Arg-Pro, flanqued by positively charged amino acids [1]. The first AT-hook (DBD1) presents a significant difference, since the first Pro is substituted by Gly. The other two AT-hooks (DBD2 and DBD3) only differ in the nature of the basic amino acids present next to the conserved core. Thus, the affinity of short peptides (10–11 amino acids) for DNA is similar both for DBD2 and DBD3 [11]. On the other hand, in the whole protein, the affinity of DBD2 for DNA is higher [1], [10], [11], which has been explained by the presence of additional basic amino acids in neighbor regions of the protein and which are missing in DBD3 [1]. In our studies we have used a peptide with nine amino acids identical to those found in DBD3 and similar to those found in DBD2. Thus, our results are relevant for the interaction with DNA of both core sequences of DBD2 and DBD3.

## Results and Discussion

As shown in the Methods section our crystals contain four complexes which are very similar. The geometry of the duplex and the central sequence of the AT-hook (PRGRP) are practically identical in the four complexes. On the other hand the external AT-hook terminal amino acids vary in each of the four complexes present in our crystal. Some of them form crosslinks with neighbor duplexes. In Figure 1 we present one of the complexes (oligonucleotide chains C, D and peptide M). The DNA duplex has a clear bend, localized in the region of base pairs 5 and 6. It determines an angle of 24° between the lower five bases and the upper seven bases of the duplex. This local bending may contribute to the overall bending detected when the whole protein is associated with DNA [12]. The DNA duplex has two AATT regions. As shown in Figure 1, the minor groove in one of them is occupied by the AT-hook, whereas the other region shows a clear spine of hydration, typical for the AATT sequence [13], [14]. The minor groove is wide in the region occupied by the AT-hook, with phosphate-phosphate distances in the range 12–13 Å. In the upper region the minor groove is rather narrow, with phosphate-phosphate distances about 9–9.5 Å. Bending of the DNA is probably due to the presence of the AT-hook, which distorts the minor groove. Packing interactions between duplexes may also have an influence [15].

The dodecamer we have used has eight consecutive A·T base pairs, so that the AT-hook might be positioned in several places. However it is clearly associated with one of the AATT regions and occupies fully its minor groove. Interactions of the guanidinium groups of both arginines 36 and 38 extend further into the neighboring base pairs. This fact excludes the possibility of finding two AT-hooks associated with our dodecamer sequence. The position we have found for the AT-hook also confirms previous studies [16], [17], which indicated that the AATT sequence is a favored site of interaction, whereas the TTAA sequence in our dodecamer does not appear to be appropriate, according to the previous studies mentioned. Optimal van der Waals interactions of the AT-hook with adenines appear to be important for such specificity [10].

The electron density map of the central region of the AT-hook is shown in Figure 2. It is clear that the extended Arg-Gly-Arg chain is tightly bound to the minor groove, but individual atoms are not visible. However the protruding carbonyl oxygen atoms of the main chain are clearly visible and determine the overall geometry of the amino acids. A clear hydrogen bond of the main chain NH group of Arg-38 is formed with one thymine oxygen. An additional weaker bifurcated hydrogen bond is formed by the NH group of Gly and two thymine oxygen atoms from the opposite strands of the duplex, as shown in Figures 1b and 2. Another view of the complex is presented in Figure 3. The guanidinium group of Arg 38 is clearly apparent in the electron density map and forms multiple hydrogen bonds, with one thymine, one sugar oxygen and the first water in the neighbor spine of hydration. On the other hand the guanidinium group of Arg 36 appears only as a small blob in the electron density map and cannot be accurately positioned. It has multiple possible interactions with neighboring bases and it may have several alternative orientations. An additional stabilizing factor of the AT-hook is the presence of two water bridges, shown in Figure 1a. These water molecules form bridges between the carbonyls of Arg 38 and Pro 39 and between Arg 36 and Pro 35. We also enclose a video (Video S1) as supporting information.

A comparison with the NMR structure [10] is shown in Figure 4. The overall conformation is rather similar, but there are two significant differences. In particular the conformation of the chain next to the central glycine is different. Our X-ray structure enters more deeply into the minor groove and has a different orientation of the main chain NH groups, which form hydrogen bonds with thymines in the DNA. The presence of such hydrogen bonds was postulated in the pioneering work of Reeves and Nissen [1]. An additional difference is the position of the guanidinium group of Arg 36, which in the X-ray structure is not uniquely positioned. However the main lesson of our work is that the AT-hook can bind to a strongly distorted DNA molecule, whereas most models of interaction assume that the B form of DNA remains practically unchanged upon binding [11], [18].

As a final point we may comment on what structural effect migth have the methylation of arginine in AT-hooks. It is known that tumour cells contain methyl-arginine in different positions of HMGA1, as reviewed by Zhang and Wang [19]. The most common site of methylation occurs in the first AT-hook [20], which has a different sequence than the DBD3 we have studied. Methylation has no direct influence on the formation of hydrogen bonds by the guanidinium group of arginine, but the presence of the methyl group would probably diminish the affinity of the AT-hook with DNA. As shown in Fig. 5, the methyl group will turn out to be in an external position if the hydrogen bonds between DNA and the guanidinium group are maintained. Contact of the hydrophobic methyl group with the solvent is in principle a destabilizing effect.

## Materials and Methods

The oligonucleotides and the peptide used in this study were prepared by conventional chemical synthesis. Crystals were obtained by vapor diffusion at 4°C using 5 mM MgCl_2_, 25 mM ammonium acetate, 25 mM Tris-HCl pH 7,5, 5% MPD (2-methyl-2,4-pentanediol) and 0.25 mM NiCl_2_. The concentrations of DNA and peptide were 0.2 mM and 0.4 mM respectively, with a 1∶2 ratio of DNA duplex to peptide. Crystals were obtained by increasing the concentration of MPD up to 48%. Diffraction data were collected at 110 K on beam line BM16 at the ESRF and processed with HKL2000 [21]. The structure was solved by molecular replacement with the program AMoRe [22], refined with REFMAC [23] and validated with 3DNA [24]. A bent DNA dodecamer (NDB code: BDL001) was used as a model in molecular replacement. Some of the amino acid residues showed very poor electron density. In particular Arg 33 could not be located in two of the complexes. Final refinement statistics and crystal data are given in Table 1. Attempts to obtain suitable crystals with many different oligonucleotides and AT-hook regions of HMGA1a have not been succesful so far.

The crystals we have obtained have a unique unit cell, with four crystallographically independent complexes. The geometry of the unit cell is strongly related to the typical P2_1_2_1_2_1_ structure of DNA dodecamers, described for the first time by Dickerson and co-workers [25]. The sides of our P2_1_ unit cell coincide with the diagonals of the P2_1_2_1_2_1_ crystals, so that our cell is equivalent to two unit cells of the Dickerson structure. A scheme of the asymmetric unit is given in Figure 6.

Figures have been prepared with either Pymol (available at http://www.pymol.org/) or with Cerius 2 (Accelrys Inc. San Diego, CA, USA).

### Accession code

Protein data bank: Coordinates have been deposited with accession code 3UXW.

## Supporting Information

Video S1 **The video presents details of the structure of the AT-hook/DNA complex viewed from different angles and at different magnifications.**
(MP4)
